# Modelling cost benefit of community-oriented primary care in rural South Africa

**DOI:** 10.4102/phcfm.v12i1.2225

**Published:** 2020-03-05

**Authors:** Rod Bennett, Tessa S. Marcus, Geoff Abbott, Jannie F. Hugo

**Affiliations:** 1 Department of Family Medicine, School of Medicine, Faculty of Health Sciences, University of Pretoria, City of Tshwane, South Africa

**Keywords:** Community-oriented primary care, Rural health, Primary healthcare, Benefit-to-cost ratio, Mining communities

## Abstract

**Background:**

Globally, rural populations have poorer health and considerably lower levels of access to healthcare compared with urban populations. Although the drive to ensure universal coverage through community healthcare worker programmes has shown significant results elsewhere, their value has yet to be realised in South Africa.

**Aim:**

The aim of this study was to determine the potential impact, cost-effectiveness and benefit-to-cost ratio (BCR) of information and communications technology (ICT)-enabled community-oriented primary care (COPC) for rural and remote populations.

**Setting:**

The Waterberg district of Limpopo province in South Africa is a rural mining area. The majority of 745 000 population are poor and in poor health.

**Methods:**

The modelling considers condition-specific effectiveness, population age and characteristics, health-determined service demand, and costs of delivery and resources.

**Results:**

Modelling showed 122 teams can deliver a full ICT-enabled COPC service package to 630 565 eligible people. Annually, at scale, it could yield 35 877 unadjusted life years saved and 994 deaths avoided at an average per capita service cost of R170.37, and R2668 per life year saved. There could be net annual savings of R120 million (R63.4m for Waterberg district) from reduced clinic (110.7m) and hospital outpatient (23 646) attendance and admissions. The service would inject R51.6m into community health worker (CHW) households and approximately R492m into district poverty reduction and economic growth.

**Conclusion:**

With a BCR of 3.4, ICT-enabled COPC is an affordable systemic investment in universal, pro-poor, integrated healthcare and makes community-based healthcare delivery particularly compelling in rural and remote areas.

## Introduction

In spite of substantially higher per capita government spending on health in South Africa, including extensive investment in primary care and the treatment of infectious diseases, there is no correspondingly significant improvement in public health when compared with that of global upper-income and middle-income countries.^1^ With the current trajectory, the country is not on course to achieve the 2020 or 2030 SDG-3 targets.^2^ Community-based healthcare, although a prioritised government policy, remains a reactive and haphazard facility add-on, with less than optimal implementation fidelity,^3^ especially in respect of service integration, project duration, community health worker (CHW) training and conditions of employment. Almost 10 years into primary healthcare (PHC) re-engineering, the reform has yet to go to scale. This, despite tested approaches on how to execute scale-up, including information and communications (ICT) enabled COPC.

### Community-oriented primary care and information and communications technology

In COPC, CHWs work full time as part of the clinical healthcare service team and are trained continuously at the workplace, using a curriculated learning programme that covers the main causes of morbidity and mortality.^4,5^ AitaHealth™, a custom-built smart phone application and web platform developed by the University of Pretoria with private and public sector partners, is used by CHWs to register and record the health status of individuals. Supported by complex algorithms, the app also provides them with decision-support, including being able to have real-time service or clinical advice and assistance; assistance with work scheduling and general planning; effective record-keeping and easily accessible data to support information and service continuity. Through the web platform, data are available to supervisors and clinicians to support the planning and management of individual and household health promotion, disease prevention and patient care as well as CHW performance and capacity development. Aggregated and anonymised, these are available to system managers for medium- and long-term reporting and resource planning. Population-based spatially defined service workload and resource needs are provided using a customised integrated health system planning toolkit (iHSP) to enable geographically defined, facility-linked healthcare service delivery to and from people at their homes.

## Cost benefit

Rural populations on all continents and in all countries have poorer health and considerably lower levels of access to healthcare facilities compared with urban population.^6^ The global drive to extend healthcare services to people at their homes through CHW programmes has shown significant results. That said, there is still a need to determine the extent to which improved health literacy and prevention, early detection and treatment support provided through ICT-enabled COPC could reduce morbidity and mortality, and impact poverty.

To better understand the worth, sustainability and comparative benefits of ICT-enabled COPC in a rural and remote context, and as a way of further contributing to the existent body of evidence for community-based healthcare services,^7,8^ this article models the potential impact, cost-effectiveness and benefit-to-cost ratio (BCR) of its implementation in the Waterberg district of Limpopo province in South Africa.

## Setting

The socio-economic indicators for this rural mining district show a very poor population living in poor circumstances with few opportunities.^9^ The local economy is dominated by mining (48.5%) that, together with community services (15.1%) and trade (11.2%), accounts for over three-quarters of all economic activity.^10^ Two in five households (40.9%) are headed by women. Although the unemployment rate is relatively low (13% in 2016), it has to be understood in the context of low formal labour force participation and absorption rates (48.6% and 39.3%, respectively, in 2017) that adversely affect young people (51% unemployed) and women in particular. Most adults have not matriculated from school (72.4% – 2016) and very few (9%) have post-school education. Much of the population (85%) live in formal dwellings, although there are low levels of connected sewerage (56.2%) and 74.1% do not have piped water in their dwellings.^11^

In terms of population health,^12^ Waterberg has an estimated 63 847 people living with human immunodeficiency virus (HIV) with an antenatal care HIV prevalence rate of 25.8% (in 2015). During 2011–2015, HIV/AIDS and tuberculosis (TB) accounted for 30.4% of young adult (aged 15–24 years) and 38.3% adult (aged 25–64 years) deaths. Notwithstanding being amongst the best performing districts in the country in terms of adult TB treatment initiation, Waterberg is ranked amongst the 10 worst performing districts in respect of TB death rate, adult and child HIV treatment compliance and viral load suppression at 12 months (80.8%) (Table 1).

**TABLE 1:** HIV and TB detection and treatment – Waterberg district, Limpopo (2016/2017).

**HIV** HIV prevalence 27.0% HIV testing coverage 45.3% Adult viral load completion rate 65.9% Child viral load completion rate 56.6% Child viral load suppressed 56.5% Adult % on ART after 12 months 67.2% **TB** TB death rate 14% (Limpopo 11.9%, South Africa 1.6%) Lost-to-follow-up (LTFU) 7.4% Treatment success rate 71.3% (Limpopo 76%) Screened in facility rate 37.8% (Limpopo 56%)

*Source:* Massyn N, Padarath A, Peer N, Day C. District health barometer 2016-17 [homepage on the Internet]. Health Systems Trust, Section 20 - Limpopo. c2017 [cited 2019 June 20]. p. 554–611. Available from: http://www.hst.org.za/publications/Pages/District-Health-Barometer-201617.aspx

HIV, human immunodeficiency virus; TB, tuberculosis; ART, antiretroviral therapy.

In terms of non-communicable diseases (NCDs), there is low cervical cancer screening coverage (58.2% of women aged 30+ years) and the incidences of hypertension (12/1000 pop) and diabetes (2.4/1000 pop) are not matched by effective treatment responses.

## Method

### Study design and approach

The study was designed to articulate the additional resources necessary to integrate a full ICT-enabled COPC service into the current district health service. It assumed that the current staffing and infrastructure would continue to deliver in-facility care but all CHWs, team leaders (TLs), clinical supervisors and care coordination staff would be added. The analysis was population-based, and the service demand was derived from risk-adjusted workload using the government’s primary care re-engineering best practice profile of contacts by condition. In addition to condition-specific contacts, the CHW workload included ensuring every household is visited annually, and routine visits involve health promotion and disease prevention, treatment-adherence support, rehabilitation and palliative care support through home-based care (HBC). The estimates of lives saved were based on condition-specific outcomes in the literature available but without adjustment for the gain of utility from years of avoided illness or disability. Staff cost was based on current government policy and occupation-specific dispensations for each of the staff cadres and was discounted at the net of growth and inflation. Equipment, vehicle and material costs were at current market values.

### Modelling assumptions

The following assumptions were used to inform the modelling: condition-specific effectiveness, population size and characteristics, health-determined service demand, resources and cost of delivery, cost-effectiveness and cost benefits.

#### Condition-specific effectiveness

Based on the evidence of the impact of well-managed community-based CHW support and care, the following ranges of impact were applied to the model (Table 2). These are considered conservative given available, albeit limited, evidence of the extent to which community-based healthcare could impact on outcomes (Table 3).

**TABLE 2:** Anticipated potential impact effectiveness (lives saved) for primary conditions.

Condition	Lower bound (%)	Expected benefits (%)	Upper bound (%)	Basis of Approach
Maternal Health	17	33	50	Upper bound set as observed in literature
Infant Health	9	18	27	Upper bound set as observed in literature
Child health	14	28	42	Upper bound set as observed in literature
HIV care and treatment	18	37	55	Lower, full viro suppr, upper max incr. on treatment
TB care and treatment	18	37	57	Upper bound increased case detection rate
NCD	6	12	18	Combined mid -range weighted by prevalence
Acute care	5	10	15	As investment case

NCD, non-communicable disease.

**TABLE 3:** Community-based healthcare effectiveness for primary conditions.

Source	Condition/issue/activity	Impact
Lehmann and Sanders^7^	Exclusive breastfeeding at 6 months	Doubled, from 15% to 30%
	Asthma symptoms screening	Reduced urgent cases by 75%
	Undernourished children	81% adequate weight gain over 2 years
	Infant mortality and diarrhoeal deaths	50% reduction – 130 to 64/1000
	Maternal mortality	50% reduction – 596 to 246/100 000
	Immunisation coverage	Increased between 70% and 100%
Van Rooyen et al.^14^	Point of care CD4 testing and lay councillor follow-up	91% uptake of HIV testing and almost universal linkage to HIV care and ART initiation
Moyo et al.^15^	Screening for TB where all participants were symptomatic and lived within 2 km of a primary clinic	Increased case finding by 2.6 times and first investigation for 77% of smear-positive participants
Ndou et al.^16^	Hypertension control	CHW support at home increased control by 63%

HIV, human immunodeficiency virus; TB, tuberculosis; CHW, community health worker; ART, antiretroviral therapy.

#### Population size and characteristics

The iHSP model uses Statistics South Africa’s (StatsSA) small area layer (SAL) data set. Each SAL is linked to the nearest PHC facility using a geographical information system (GIS). This ‘nearest neighbour’ approach was considered to be robust because 92.8% of households used the nearest health facility.^13^ The catchment population and its characteristics (age, gender, households and income profile) for each PHC facility are thus defined by the sum of the SAL population profiles.

Statistics South Africa’s full national data^17^ were used to calculate income thresholds for each of the income quintiles. These were used to determine the number of households in each of the income quintiles in each small area. The analysis focussed on quintiles 1 to 4, assuming quintile 5 population was insured privately. Population growth was applied by district, based on StatsSA mid-year estimates (2011–2018).^18^ Income was discounted using the net value of growth versus inflation, as increase in wealth and wages was eroded by inflation.

#### Health determined service demand

National mortality and morbidity rates hide a significant variation in rates by income level, with the risk of under-5 mortality in low- and middle-income countries being twice as high for children in the poorest households compared with those in richest.^19^ The mortality ratio between the poorest and the richest households is 2.78 for communicable diseases in general, 5.9 for maternal deaths, 4.9 for child deaths, 3.3 for diabetes mellitus, 9.1 for TB and 2.3 for cardiovascular disease.^20^

Service demand, as reflected in the number of visits required, is adjusted for relative burden of disease using income and mortality rates. Baseline mortality is calculated in the tool from mortality rates for each health condition. Because mortality rates vary with income, the mortality rates for the selected quintiles were further adjusted in the analysis.

#### Resources and costs of delivery

The costing is based on the annual full operational cost of the community-based teams. Costing of CHWs and HBCs was performed in line with the government’s minimum wages (2019 – R20.00 per hour or R3500 per month) for full-time employment (a 40-h working week) with paid and sick leave benefits.

Community health workers and TLs (staff nurses and equivalent) were managed by a coordinator (professional nurse or equivalent) at the health facility and each sub-district had a contracting unit manager (clinician). Equipment costs were annualised and included maintenance and replacement. Community health worker kit included replacement of equipment and replenishment of consumables. Transport costs were included for TL and a clinical supervisor to carry out twice-weekly in-field supervision and twice-monthly field team visits, respectively. Community health workers were provided with uniforms and patient information leaflets for each of the seven conditions. Each team member was given two cycles of 16-day in-service training for a year. Marginal costs of space and other resource utilisation incurred at facility level were not included in the modelling.

For communication and data management, each CHW and TL had either a smart phone or tablet. Each clinic was provided with general consumables as well as a computer, a printer, Wi-Fi and uninterruptible power supply (UPS).

From experience, data collected on the AitaHealth^TM^ platform lead to a multiplicity of savings and service efficiencies when compared with paper-based systems. Information and communications technology removes the need for data clerks, as well as filing cabinets and space for storage. It allows CHWs to work remotely. This means that they can clock in and out without incurring out-of-pocket expenses on transport or system loss to work time costs. Through real-time data, TLs have visibility of individual and team performance, creating efficiency in planning and in-field service support. Because healthcare workers are able to update, send, store and retrieve existing records, ICT supports information continuity, streamlines household visits and contributes to quality of care. Thus, whilst the time spent by a CHW visiting a household (visit time) in both systems depended on individual multi-morbidity and the number of people requiring healthcare, ICT-related savings reduced the actual time allocated per service for a single condition. Covering all hardware and software costs and overheads, ICT yields an overall 15% savings on paper-based systems.

To see whether the benefits accruing from that investment warrant expenses, for this study the entire community-based service was considered to be an additional cost, irrespective of the source of funding. We assumed a steady state where if the interventions and investments to save lives were not sustained, then morbidity and mortality would revert to pre-intervention levels. Conservatively, we ignored secondary positive knock-on effects of reduced morbidity and the future lives saved through early detection and treatment, reduction in infections and improved health literacy in the population.

The costs of CHW interventions in relation to each condition were proportionally based on the proposed number of visits related to that condition. Annual revisits to all households were critical for the expected outcomes as they enabled healthcare services to update and respond to demographic, social and healthcare changes.

#### Cost-effectiveness

Life years saved are measured as the difference between average age of the affected population for each condition, namely, people living with HIV, TB and adults (32 years, 15–49); pregnant women (25 years, 15–35), stillbirths and infants (0 years); children (3 years, 0–6); NCD (45 years, 40–50) and national life expectancy (60 years, 56–64). Cost-effectiveness is calculated as the cost per life year saved for each condition.

The calculation of cost-effectiveness was based on the full incremental cost of adding community-based services to facility services as described above.

We also explored the minimum impact thresholds that would make the interventions not very cost-effective (using both gross domestic product [GDP] per capita and average income).

#### Cost benefit

The benefits of intervention were measured in terms of the discounted earnings for the remainder of the lives saved by population group and condition as well as the earnings of the CHWs employed. Both individual benefits and gains to the economy were included. As the population served was relatively poor, it was assumed that they would not accrue savings but would substantially return all of their earnings to the economy through the purchase of goods and services. Adjustments were made for tax (18% standard rate), underlying level of unemployment, children’s schooling^21^ and child support grants.^22^ For poverty reduction, tax was deducted but not the cost of schooling and support grants. For GDP growth, tax was not deducted (because it was transferred to the state’s benefit) but the cost of schooling and support grants were deducted (as costs to the state).

### Ethical considerations

Ethics clearance was obtained from the Faculty of Health Sciences Research Committee (University of Pretoria) (Reference No.: 102/2011).

## Results

The resource needs and costs are shown in Tables 4 and 5. Note that numbers of CHWs and HBCs are the actual numbers employed according to the model. The costs make allowance for part-time CHW employment in sparsely populated areas.

**TABLE 4:** Summary of populations served and resource needs by proximity to the nearest primary healthcare facility.

Item	Proximal: <2 km	Distal: >2 km, >200 p/sq km	Remote: >2 km, <200 p/sq km	Total
Vists per capita	2,96	2,98	2,95	-
Households per CHW	269	249	50	-
Pop per CHW	1014	981	156	-
Teams/TL	54	32	17	103
Actual HBC	67	38	77	182
Actual CHW	360	205	414	979
CSPC per CHW	3001	2926	461	-
Time per CSPC	28	28	30	-
Working hours	7	7	7	-
Working days	180	180	180	-
CSPC	1 080 229	599 506	190 748	1 870 482
Population	364 919	200 887	64 759	630 565
Households	96 886	50 943	20 517	168 345

CHW, community health worker; HBC, home-based care; PHC, primary healthcare; CSPC, condition-specific person contacts; TL, team leader.

**TABLE 5:** Summary of additional costs by area of expenditure for delivery of community-oriented primary care.

Cost item	Number	Total	% of total
CHW FTE	643	27 015 933	28
HBC FTE	120	5 030 067	5
TL	103	19 551 511	20
Facility link to care (LtC)	103	5 132 400	5
Clinical associate	63	24 139 080	25
Family physician	6	2 912 328	3
TL-PPT	49	1 871 371	2
Clinical supervisor	49	650 302	1
Computers and printers	63	556 920	1
Wireless fidelity (Wi-fi), UPS	63	255 969	0
Cell phone for CHW’s	1283	1 539 840	2
Mobile system	1283	2 309 760	2
Tablets for OTL	103	123 720	0
CHW kit	1161	1 741 500	2
Uniform	1161	298 841	0
Leaflets	1161	2 043 360	2
CHW – materials	1104	1 104 000	1
TL – materials	122	122 200	0
Overall total	-	96 399 102	-

CHW, community health worker; HBC, home-based care; PHC, primary healthcare; FTE, full-time equivalents; UPS, uninterruptible power supply; TL, team leader; TL-PPT, team leader - planned patient transport.

It was calculated that 122 teams (122 TLs, 979 CHWs, 182 HBCs) would be needed to deliver a full package of community-based healthcare to 630 565 people eligible for such services in Waterberg.

## Impact

In 2016, the all-cause mortality in Waterberg district and Limpopo province was 4899 and 45 578, respectively.^23^ For the conditions covered by CHWs in the community, we calculated total mortality in Waterberg to be 4279, of which 932 (22.6%; 490–1472) deaths could be avoided annually at scale. This equated to 36 134 (17 854–53 278) unadjusted life years saved each year (35 877 allowing for all-cause mortality in the cohort). Table 6 shows the impact on lives and life years saved by each condition.

**TABLE 6:** Information and communications technology-enabled community-oriented primary care’s annual cost-effectiveness summary results.

Condition	Condition-specific consultations	Proportion of condition-specific consultations (%)	Proportion of total† consultations (%)	Baseline mortality	Lives saved	Life years saved	Cost per life year saved (Rand)
Maternal and neonatal health	159 048	17	10	18	7	3970	3039
Infant health	114 126	12	7	237	48	2863	3282
Child health	113 975	12	7	264	85	5120	1833
HIV care and treatment	180 453	19	11	1368	506	14 171	807
TB care and treatment	153 268	16	10	146	54	1885	5209
NCD	177 720	19	11	1831	220	7689	1466
Acute care	52 967	6	3	416	12	437	8826

ICT, information and communications technology; COPC, community-oriented primary care; HIV, human immunodeficiency virus; TB, tuberculosis; NCD, non-communicable diseases.

† , Total of comprehensive healthcare consultations includes social support along the health–disease continuum.

## Cost and cost-effectiveness

The annual cost of community-level healthcare delivery was R96.4 million, including R10.1m for non-staff costs. This translates to a cost per capita of population served as R170.37 and a cost per life year saved as R2668 (R1809–R5399). Table 6 shows details of cost-effectiveness according to condition.

Based on GDP per capita (R99 169), the full, World Health Organisation (WHO)-aligned cross section of interventions delivered through community-based services were extremely cost-effective. They were extremely cost-effective, measured against the lower cohort average household income (R79 524); and even if the effectiveness of the interventions for all conditions was adjusted downwards, the cost per life year saved matched the GDP per capita and average household income at 0.60% and 0.75%, respectively. With 1% improvement in performance, the cost per life year saved was R56 293.

## Cost benefit

The cost-effectiveness results show substantive savings, even though they do not include the net savings in the health system that are realistically expected to arise because of avoided hospital admission, reduced primary care attendance and reduction in usage of drugs from avoided infections.

Savings from HIV and TB care and treatment would amount to a minimum of R39.4m. This was determined using the SAMRC IC calculation of 2.2 times the cost of treatment and medications (2.2 × 20.3% of 88.3). It does not account for secondary impacts of averted infections that could be anticipated in medium to long term.

At scale, a reduction of 110 664 in PHC clinic attendance, 4729 in hospital admissions and 23 646 in outpatient attendance are expected to give a net average annual saving of R120m (R60.1–R179.8m). The saving to Waterberg district is estimated at R63.4m per annum. Moreover, R42.3m paid in wages per annum to CHWs and TLs would contribute directly to poverty reduction in their households.

In terms of adult and child poverty reduction, the present value of earnings in community Q1–Q4 households from lives saved would increase on average by R40.7m (R20.1–R60.4m). If the discount rate is adjusted from the 0.72% net growth value to 3% and 6%, the amount would be R30.3m and R21.3m, respectively.

On reaching scale, community-based healthcare services would contribute an average of R492m annually towards poverty reduction and economic growth in the district. The value of the contribution of these services towards cumulative poverty reduction would be R4.9 billion over a 10-year period or R4.1bn if discounted at 3% or R3.4bn if discounted at 6%. These amounts would contribute to the local economy as a part of provincial or national GDP.

The combined annual contribution of team wages and community household earnings to the local economy would be R83.1m (R62.4–R102.7) or R72.6m and R63.6m, respectively, if the discount rate is adjusted to 3% and 6% from the 0.72% net growth value.

Overall, the annual sum of all the benefits divided by the total additional costs for delivering community-based healthcare using an ICT-enabled COPC approach produced a BCR of 3.4.

## Discussion

The historical and ongoing challenge of providing allopathic healthcare services to rural, often small, scattered and physically remote settlements of people has been difficult to overcome globally.^6^ In South Africa and in many other parts of the world, this is partly because of the extra costs in time and money required to reach them effectively and the difficulties of providing the appropriate generalist and specialist skills and services mix to these communities.^24^ In part, it is also because of the manner in which the medical industrial complex has restructured healthcare provisions in the second half of the 20th century.^25^ Hart’s 1971 articulation of the law of inverse care^26^ is particularly salient to the present healthcare crisis being faced by South Africa. The effects of poorer healthcare provisions to people with greater needs make a compelling case for a more effective healthcare system in the Waterberg district.

To illustrate the significance of a rural setting, we compare the study district with an urban metropolitan municipality comparator (Ekurhuleni). Household size in Waterberg ranges from 3.2 to 4.0 persons, whereas in Ekurhuleni, it ranges from 2.6 to 2.9 persons. The proportion of urban and rural SALs with less than 100 households in Waterberg ranges from 42% to 83% compared with 25%–67% in Ekurhuleni. The number of households per CHW ranges from 46 to 53 (remote) in Waterberg and from 230 to 385 (urban) in Ekurhuleni. The ratio of total CHWs employed as full-time equivalents (FTE) is 1.5 in Waterberg and 1.1 in Ekurhuleni. The proportion of households living in extreme poverty (less than $2 per person per day) is 43% in Waterberg and 30% in Ekurhuleni.

There are always choices to be made in healthcare provisions. In the 21st century, conceptual and practical advances change the way healthcare services could be organised and delivered. Modelling the extension of healthcare through community-based services using 21st-century, ICT-enabled COPC shows that it is possible to provide better and more cost-effective healthcare with greater impact on individual, family and community well-being. Moreover, this approach would be particularly beneficial for rural, remote and marginal populations that are underserved by the existing system of fragmented and under-resourced facility-based services.

Although healthcare delivery in community-based services is rendered primarily by CHWs, COPC assumes that they do this under clinical guidance; that they are supported in the delivery of services by ICT; that their competencies are developed through continuous workplace learning and that they are integrated into the healthcare system. These assumptions are critical to the case for cost-effectiveness in healthcare not only because they have a direct bearing on the quality of services rendered by CHWs but also because they talk to the importance of care coordination to produce the best possible patient outcomes.

Creating community-based services triggers the need to develop a system of integrated care from people in their homes to and from facilities. Although not included in this analysis, making structural changes in the healthcare system, including in the scope of practice of healthcare professionals and practitioners,^27^ could generate further savings while increasing patient and societal benefits.

Notwithstanding that savings from hospitalisation were not included in this study, the analysis shows that the savings that would be realised by the district from drugs, treatment and hospitalisation exceed the cost of implementing community-based services at scale.

Apart from health benefits and efficiencies, ICT-enabled COPC is pro-poor,^28^ directly and indirectly contributing to poverty reduction through community-based services. By making home the starting and end point of healthcare service contacts, individual and household out-of-pocket expenses and income forfeiture as well as income loss would be reduced. Moreover, the extent of these savings would grow as system functioning, healthcare team proficiency and technology combine to improve health literacy and service, information and care continuity.

Community-based services in an integrated healthcare system provide regular income for CHWs who are drawn from the communities they serve. Because CHWs are predominantly female, the poverty-reduction component of the benefits is also particularly well targeted because women are more likely to use income to support their families.^29^ In addition, such work is also likely to have a direct knock-on benefit for vulnerable and female-headed households, not only because of the value of regular income but also because of the skills and competencies they acquire through their work.

While community-based services afford people the opportunity to be employed in work that is decent, their sustainability as a cost effective solution to healthcare delivery requires that issues of pay and conditions of employment are normalised. This is particularly relevant bearing in mind that ‘the purpose of minimum wages is to protect workers against unduly low pay’ and to be a policy instrument that seeks ‘to overcome poverty and reduce inequality’ between men and women, and occupations and geography.^30^

It is customary to use the GDP per capita threshold for cost-effectiveness as recommended by WHO to avoid the inference that poor people’s lives are less valuable than those of rich. However, although it is based on affordability, using the GDP per capita threshold shifts the value judgement to national levels, implying that the lives of citizens in rich countries are more valuable than those in poor countries. We consider that calculating cost benefit on this basis is unrealistic. Having introduced the metric of average annual household income for cost benefit, we also apply this to cost-effectiveness, and show that even at much lower threshold, a very small number of lives saved makes the investment cost-effective.

### Limitations

One limitation relates to health conditions. The analysis excludes violence and injuries, which account for 10% of life years lost, although community-based interventions are expected to reduce related morbidity and mortality through increased health literacy and risk awareness. It also does not take account of the benefits of recuperation and support at home. Another limitation is that the prevalence and incidence rates used are district averages, whereas there is considerable variation across communities within the district. This could be mitigated in the analysis through community-specific risk adjustment profiling, but it does not adjust for non-poverty causes of incidence, such as HIV and TB related to mining communities. The BCR is understated because it is based on an annual average cost–benefit of over 10 years, whereas the benefits would be accruing from the time the first infant death is avoided until that infant retires as an adult. Also, it is assumed that the interventions would continue to save the same number of lives over 10 years, although, in fact, the number of lives saved is likely to be greater with decrease in conditional incidences, increase in health literacy and changes in the risk profile of populations.

## Conclusion

The results of this study show that ICT-enabled COPC in a rural setting is extremely cost-effective, providing overall savings to the health system, and reducing the service burden on higher levels of care while significantly reducing poverty.

Because of the higher burden of disease, the case for community-based service delivery is especially compelling in rural and remote areas such as Waterberg (Limpopo). At an annual cost of R96m and R2668 per life year saved, it is extremely cost-effective. Set against the R155m anticipated savings of the district, it is both a cheaper and a more effective system than what is available currently. With the additional R175m in societal benefits that come from poverty reduction and an increase in GDP, it yields a BCR of 3.4.

Information and communications technology-enabled COPC is an affordable systemic investment in a universal and pro-poor, integrated healthcare system. Mainstreamed and taken to scale, the savings and improved outcomes in overall health and well-being that it generates could contribute significantly towards the national effort to achieve 2030 SDG-3 targets.

## References

[1] Doherty J Critical assessment of different health financing options in east and southern African countries [homepage on the Internet]. Regional network for equity in health in east and southern Africa. EQUINET Discussion paper 119. c2019 [cited 2019 June 20]. Available from: http://www.equinetafrica.org/sites/default/files/uploads/documents/EQ%20Disspaper%20119%20Fin%202019.pdf

[2] World Health Organisation (WHO) SDG 3: Ensure healthy lives and promote wellbeing for all at all ages [homepage on the Internet]. No date [cited 2019 June 20]. Available from: https://www.who.int/sdg/targets/en/

[3] Carroll C, Patterson M, Wood S, Booth A, Rick J, Balain S A conceptual framework for implementation fidelity. Implement Sci. 2007;2:40 10.1186/1748-5908-2-40 18053122 PMC2213686

[4] Marcus TS COPC – A practical guide. Pretoria: Minuteman Press; 2018.

[5] Bennett R, Marcus TS, Abbott G, Hugo JF (2018). Scaling community-based services in Gauteng, South Africa: A comparison of three workforce-planning scenarios. Afr J Prim Health Care Fam Med. 2018;10(1):a1748 10.4102/phcfm.v10i1.1748 PMC6018689 29943603

[6] Scheil-Adlung X (Ed.) Global evidence on inequities in rural health protection: New data on rural deficits in health coverage for 174 countries. Extension of social security series, No. 47 [document on the Internet]. International Labour Office, Social Protection Department; c2015 [cited 2019 June 22]. Available from: https://www.ilo.org/wcmsp5/groups/public/---ed_protect/---soc_sec/documents/publication/wcms_383890

[7] Lehmann U, Sanders D Community health workers: What do we know about them? The state of the evidence on programmes, activities, costs and impact on health outcomes of using community health workers [document on the Internet] School of Public Health, University of the Western Cape; c2007 [cited 2018 Aug 09]. Available from: http://www.chwcentral.org/sites/default/files/Community%20Health%20Workers-%20What%20do%20we%20know%20about%20them.pdf

[8] Daviaud E, Besada D, Budlender D, Sanders D, Kerber K 2018. Saving lives, saving costs. Investment case for community health workers in South Africa What costs and what benefits for the health sector, for the economy and for society? [document on the Internet]. South African Medical Research Council; c2018 [cited 2018 Aug 9]. Available from: http://www.mrc.ac.za/sites/default/files/files/2017-10-30/SavingLivesSavingCosts.pdf

[9] Massyn N, Padarath A, Peer N, Day C District health barometer 2016/17 [homepage on the Internet]. c2017 [cited 2019 June 20]. Available from: http://www.hst.org.za/publications/Pages/District-Health-Barometer-201617.aspx

[10] Pratt GC Limpopo economic review and outlook [document on the Internet]. Provincial government; c2017 [cited 2019 Jan 31]. Available from: http://www.treasury.gov.za/documents/provincial%20budget/2017/5.%20Guide%20to%20the%20Budget/LIM/Limpopo%20Socio-Economic%20Review%20and%20Outlook.pdf

[11] Massyn N, Tanna G, Day C, Ndlovu N District health barometer: District health profiles 2017/18 [document on the Internet]. Durban: Health Systems Trust; c2018 [cited 2019 June 20]. Available from: https://www.hst.org.za/publications/District%20Health%20Barometers/Distric%20Health%20Barometer-District%20Health%20Profiles%2020172018.pdf

[12] National Department of Health The 2015 national antenatal Sentinel HIV & syphilis survey [document on the Internet]. c2017 [cited 2019 June 19]. Available from: http://www.doh.gov.za

[13] Statistics South Africa General household survey 2015. Statistic Release P0318 [document on the Internet]. c2016 [cited 2017 Aug 24]. Available from: https://www.statssa.gov.za/publications/P0318/P03182015.pdf

[14] Van Rooyen H, Barnabas RV, Baeten JM, et al High HIV testing uptake and linkage to care in novel program of home-based HIV counselling with facilitated referral in KwaZulu-Natal, South Africa. J Acquir Immune Defic Syndr. 2013;64(1):e1–e8. 10.1097/QAI.0b013e31829b567d 23714740 PMC3744613

[15] Moyo S, Verver S, Mahomed H, et al Age related tuberculosis incidence and severity in children under 5 years of age in Cape Town, South Africa. Int J Tuberc Lung Dis. [serial online]. 2010 [cited 2019 June 30];14(2):149–154. Available from: https://www.ingentaconnect.com/content/iuatld/ijtld/2010/00000014/00000002/art00005 20074404

[16] Ndou T, Van Zyl G, Hlahane S, Goudge J A rapid assessment of a community health worker pilot programme to improve the management of hypertension and diabetes in Emfuleni sub-district of Gauteng Province, South Africa. Global Health Action. 2013;6:1 10.3402/gha.v6i0.19228 PMC3556684 23364086

[17] Statistics South Africa (StatsSA) Personal communication (content only provides 2001 census data) [homepage on the Internet]. 2013 [cited 2019 June 10]. Available from: www.statssa.gov.zadatabase&site

[18] Statistics South Africa (StatsSA) Mid-year population estimates 2011–2018 [homepage on the Internet]. 2018 [cited 2019 June 26]. Available from http://www.statssa.gov.za/?p=11341

[19] Chao F, You D, Pedersen J, Hug L, Alkema L National and regional under-5 mortality rate by economic status for low-income and middle-income countries: A systematic assessment. Lancet Glob Health. 2018;6(5):e535–e547. 10.1016/S2214-109X(18)30059-7 29653627 PMC5905403

[20] An American Health Organization, World Health Organization Health in the Americas [homepage on the Internet]. c2017 [cited 2018 June 22]. Available from: https://www.paho.org/salud-en-las-americas-2017/

[21] UNICEF Education budget for South Africa 2017-18 [homepage on the Internet]. n.d. [cited 2019 June 25]. Available from: https://www.unicef.org/southafrica/resources_20884.html

[22] South African Social Security Agency (SASSA) In 2018 child support grant increased to R410 per month for children under 18 [homepage on the Internet]. [cited 2019 June 25]. Available from: https://www.sassa.gov.za/Pages/Child-Support-Grant.aspx

[23] Statistics South Africa (StatsSA) Mortality and causes of death in South Africa, 2016: Findings from death notification. Stastistical Release P0309.3 [document on the Internet]. c2018 [cited 2019 June 25]. Available from: http://www.statssa.gov.za/publications/P03093/P030932016.pdf

[24] Australian College of Rural and Remote Medicine Cairns consensus statement on rural generalist medicine. Improved health for rural communities through accessible, high quality healthcare [document on the Internet]. c2014 [cited 2019 June25]. Available from: http://www.acrrm.org.au/docs/default-source/documents/about-the-college/cairns-consensus-statement-final-3-nov-2014.pdf

[25] Gale AH The hospital as a factory and the physician as an assembly line worker. Mo Med. [serial online]. 2016 [cited 2019 Jan 31];113(1):7–9. Available from: https://www.ncbi.nlm.nih.gov/pmc/articles/PMC6139751/PMC6139751 27039480

[26] Hart JT The inverse care law. Lancet. 1971;297(7696):405–412. 10.1016/S0140-6736(71)92410-X 4100731

[27] McPake B, Edoka I, Witter S, et al Cost-effectiveness of community-based practitioner programmes in Ethiopia, Indonesia and Kenya. Bull World Health Organ. 2015;93(9):631–639A. 10.2471/BLT.14.144899 26478627 PMC4581637

[28] Bennett S, Gilson L Health financing: Designing and implementing pro-poor policies [document on the Internet]. c2001 [cited 2019 June 28]. Available from: http://www.heart-resources.org

[29] Yoong J, Rabinovich L, Diepeveen S The impact of economic resource transfers to women versus men: A systematic review. Technical report [document on the Internet]. c2012 [cited 2019 June 28]. Available from: https://eppi.ioe.ac.uk/cms/Portals/0/PDF%20reviews%20and%20summaries/Economic%20transfers%202012Yoong.pdf?ver=2012-01-13-101615-493

[30] International Labour Organisation (ILO) Minimum wage policy guide: Minimum wages: Definition and purpose [homepage on the Internet]. c1996–2019 [cited 2019 June 28]. Available from: https://www.ilo.org/global/topics/wages/minimum-wages/definition/WCMS_439072/lang--en/index.htm

